# Comparative Prognostic Performance of Inflammatory and Immunonutritional Indices in Acute Pancreatitis: A Head-to-Head Cohort Study

**DOI:** 10.3390/jcm15197495

**Published:** 2026-09-26

**Authors:** Soner Yeşilyurt, Adem Şentürk, Türker Demirtakan, Recep Yılmaz Bayraktarlı, Mehmet Atilla Uysal, Savaş Öztürk

**Affiliations:** 1 Department of Internal Medicine, Istanbul Taksim Training and Research Hospital, University of Health Sciences, Istanbul 34433, Türkiye; 2 Department of Surgical Oncology, Sakarya University Training and Research Hospital, Sakarya 54100, Türkiye; dr.adem.senturk@gmail.com; 3 Department of Emergency Medicine, Istanbul Taksim Training and Research Hospital, University of Health Sciences, Istanbul 34433, Türkiye; turkerdemirtakan@gmail.com; 4 Department of Radiology, Prof. Dr. Cemil Taşçıoğlu City Hospital, University of Health Sciences, Istanbul 34384, Türkiye; reyilbay@gmail.com; 5 Department of Chest Diseases, Faculty of Medicine, Istanbul Nişantaşı University, Istanbul 34398, Türkiye; mehmetatilla.uysal@nisantasi.edu.tr; 6 Division of Nephrology, Department of Internal Medicine, Istanbul University Istanbul Faculty of Medicine, Istanbul 34093, Türkiye; savasozturkdr@gmail.com

**Keywords:** acute pancreatitis, CALLY index, GINI, neutrophil-to-lymphocyte ratio, inflammatory biomarkers, immunonutritional indices, composite outcome, BISAP, risk stratification

## Abstract

**Background/Objectives:** Simple admission laboratory indices have been proposed for early risk stratification in acute pancreatitis (AP), but their relative performance and their value beyond established severity assessment are rarely examined directly. We performed an exploratory head-to-head comparison of eight admission inflammatory and immunonutritional indices, including the Global Immune-Nutrition-Inflammation Index (GINI), for a composite adverse in-hospital outcome. **Methods:** This single-center retrospective study (2020 to 2026) analyzed the index admission of each adult hospitalized with AP (one observation per patient). Eight indices were evaluated (NLR, CAR, PIV, PNI, HALP, CALLY, CCR, GINI). The primary endpoint was a composite of in-hospital death, intensive care unit (ICU) admission, or prolonged hospitalization (a length of stay of at least 7 days). We assessed discrimination (area under the curve, AUC, with DeLong intervals and Holm-corrected comparison), age-adjusted logistic regression, calibration, bootstrap validation, and incremental value over the Bedside Index for Severity in Acute Pancreatitis (BISAP), with a patient-clustered robustness analysis. **Results:** Of 238 patients, 73 (30.7%) met the composite outcome (prolonged stay 63, ICU 15, death 2). After age adjustment, all eight indices were associated with the outcome (all *p* < 0.001). The three numerically highest were NLR (age-adjusted AUC 0.737, 95% CI 0.667 to 0.808), CALLY (0.735), and GINI (0.734), with no significant pairwise difference after correction. Each added discrimination beyond BISAP (BISAP alone AUC 0.631 vs. BISAP plus CALLY 0.752; likelihood ratio *p* < 0.001). GINI was numerically higher but not significantly better than its components (CAR 0.710, PIV 0.708). Models were well calibrated (calibration slope 0.97 to 1.00). **Conclusions:** In AP, several admission indices, led by NLR, CALLY, and GINI, gave modest, broadly comparable discrimination for a composite adverse in-hospital outcome and added information beyond BISAP. No index was superior, and the composite was driven largely by prolonged hospitalization. These exploratory findings require prospective external validation before any clinical use.

## 1. Introduction

Acute pancreatitis (AP) is among the most common gastrointestinal causes of acute hospital admission, with a global incidence of about 34 per 100,000 person-years [1,2]. Roughly one in five patients develops a moderately severe or severe course with organ failure, local complications, and increased mortality [3]. Adverse in-hospital events, namely intensive care unit (ICU) admission, prolonged hospitalization, and death, drive AP-related resource use and are directly relevant to escalation and discharge decisions [4].

A central challenge in AP is the heterogeneity of its early course, formalized by the revised Atlanta classification [5]. Established scores, the Bedside Index for Severity in Acute Pancreatitis (BISAP), Acute Physiology and Chronic Health Evaluation II (APACHE II), Ranson, and the computed-tomography severity index (CTSI), are limited in practice by their complexity, reliance on imaging, and need for 24 to 48 h to complete [6,7,8,9]. This has motivated a search for simple admission laboratory biomarkers that add information beyond established scores.

AP is increasingly viewed as a systemic disease shaped by inflammation, nutritional reserve, and immune competence [3,10], with an early pro-inflammatory phase often followed by lymphopenia and immune dysregulation [11], so that no single conventional marker captures all dimensions. Several admission composite indices from the complete blood count and basic biochemistry have therefore been proposed: the neutrophil-to-lymphocyte ratio (NLR) and pan-immune-inflammation value (PIV) reflect inflammation [12,13]; the C-reactive protein (CRP)-to-albumin ratio (CAR) and CRP-to-calcium ratio (CCR) add markers of physiological reserve [14,15,16]; the prognostic nutritional index (PNI) and hemoglobin-albumin-lymphocyte-platelet score (HALP) capture the immunonutritional status [17,18]; and the CRP-albumin-lymphocyte (CALLY) index integrates all three in a single ratio [19]. The Global Immune-Nutrition-Inflammation Index (GINI) is a higher-order composite equal to the product of CAR and PIV [20]. It has not, to our knowledge, been evaluated in AP, and whether it adds discrimination over its constituents is unknown. In AP, individual indices have been linked to severity, organ failure, or mortality [14,15,21,22,23,24,25], with CAR reported as the strongest predictor of prolonged hospitalization [24]. However, their relative performance head-to-head in one cohort, for a comprehensive composite outcome, and against an established score, is unclear.

We therefore performed an exploratory head-to-head comparison of eight admission indices (NLR, CAR, PIV, PNI, HALP, CALLY, CCR, and GINI) for a composite adverse in-hospital outcome (death, ICU admission, or prolonged hospitalization) in adults with AP, and examined whether they add discrimination beyond BISAP, whether GINI improves on its components, and how the results translate into a simple bedside score.

## 2. Materials and Methods

### 2.1. Study Design and Setting

This single-center retrospective cohort study was conducted at our tertiary referral center. Consecutive adults hospitalized with AP between 2019 and 2026 were screened; because no 2019 admission had a complete index panel, the analytic cohort spanned 2020 to 2026. The protocol was approved by the Non-Interventional Clinical Research Ethics Committee of Istanbul Taksim Training and Research Hospital (original approval: decision no. 31, 14 May 2025, ref. E-62190176-663.05-276308669; amendment extending the study period: 21 July 2026, ref. E-62190176-663.05-323433301) and conducted per the Declaration of Helsinki. Reporting follows the Strengthening the Reporting of Observational Studies in Epidemiology (STROBE) guideline [26]; the exploratory prediction components additionally follow TRIPOD + AI considerations [27]. The analyses were planned but not pre-registered. Screening covered admissions from 1 August 2019 to 20 May 2026; because no 2019 admission had a complete index panel, the analytic cohort spanned 2020 to 20 May 2026, and 2026 therefore represents a partial calendar year.

### 2.2. Patients, Independence of Observations, and Cohort Definition

Adults (at least 18 years) with a clinical diagnosis of AP by the revised Atlanta classification (at least two of acute-onset epigastric pain, lipase or amylase at least three times normal, and characteristic imaging) were eligible. A data curation flag marked a few admissions with incomplete confirmatory documentation; these were retained (the treating team diagnosed AP) and their exclusion was a sensitivity analysis. Because the source contained repeat admissions (566 from 370 patients), the primary analysis used one index admission per patient (the first qualifying AP admission). The cohort was restricted to admissions with a complete panel for all eight indices (positive PIV), yielding 238 patients (Figure 1); 132 of 370 (35.7%) were excluded for an incomplete panel and compared with those included (Supplementary Tables S1 and S2). All qualifying admissions were retained in a clustered robustness analysis.

### 2.3. Data Collection and Laboratory Timing

Demographic data, comorbidities, etiology, admission vital signs, the admission laboratory panel, in-hospital course, and length of stay (LOS) were extracted from the electronic medical record. Index components were taken from the first admission blood sample. Because exact acquisition timestamps for each analyte were not available in the de-identified dataset, we could not formally confirm that every component preceded a same-admission ICU transfer, and the term “admission-based” refers to the first available admission panel rather than a strictly time-stamped 0 to 24 h window. Because acquisition timestamps for individual analytes were not retrievable from the de-identified extract, we could not verify that every index component preceded a same-admission ICU transfer, nor that all components of a given index came from one specimen. The indices are therefore best understood as the first recorded laboratory measures of the admission, associated with the subsequent hospital course, rather than as strictly time-anchored admission predictors. We further acknowledge that the same laboratory abnormalities were visible to the treating team and may themselves have influenced decisions to escalate care or to defer discharge, so that predictor and outcome are not fully independent.

### 2.4. Inflammatory and Immunonutritional Indices

Eight admission indices were calculated for each patient using the following formulas (albumin in g/L, cell counts in ×10^9^/L, CRP in mg/L):

NLR (neutrophil-to-lymphocyte ratio) = neutrophils/lymphocytes [12].
PIV (pan-immune-inflammation value) = (neutrophils × platelets × monocytes)/lymphocytes [13].
CAR (CRP-to-albumin ratio) = CRP/albumin [14].
PNI (prognostic nutritional index) = albumin + 5 × lymphocytes [17].
HALP (hemoglobin-albumin-lymphocyte-platelet score) = (hemoglobin × albumin × lymphocytes)/platelets [18].
CALLY (CRP-albumin-lymphocyte index) = (albumin × lymphocytes)/(CRP × 10) [19].
CCR (CRP-to-calcium ratio) = CRP/calcium (mg/dL) [16].
GINI (Global Immune-Nutrition-Inflammation Index) = (CRP × neutrophils × platelets × monocytes)/(albumin × lymphocytes), which is algebraically equal to CAR × PIV [20].

For CALLY, the factor of 10 in the denominator reproduces the conventional albumin (g/dL) formulation while using albumin in g/L. GINI is not a new index: it was introduced in oncology and, as shown by its formula, is the product of two panel members (CAR and PIV) and requires CRP, albumin, and the complete blood count; its relationship to its constituents is examined directly. Because the composite indices are unit-dependent, all reported cut-offs apply to the units above and to the local assays (CRP and albumin on an automated immunoturbidimetric or colorimetric platform); values below the CRP detection limit were handled as reported by the analyzer. All indices were computed from locally measured values; in the analytic cohort the lowest observed CRP was 0.76 mg/L, no value was zero or below the analyzer detection limit, and no substitution or truncation was therefore required before logarithmic transformation.

### 2.5. Outcome Definition and the Diagnosis-Suspect Flag

The primary endpoint was a composite adverse in-hospital outcome, defined a priori as any of in-hospital death, ICU admission, or prolonged hospitalization (length of stay of at least 7 days). The 7-day threshold was selected before the final analyses on the basis of clinical convention and the prior literature [24], not from the observed distribution. ICU admission was a documented transfer to a structurally designated intensive care unit; a more inclusive definition was a sensitivity analysis. Outcome ascertainment was not blinded to the index values (a limitation). The composite was chosen because death and ICU admission, though uncommon, are decisive events a length-of-stay endpoint alone would miss; component overlap is reported. The three components were combined because each represents a failure of an uncomplicated recovery, and because death (two events) and ICU admission (15 events) alone would have been severely underpowered. We nonetheless recognize that the composite is numerically dominated by prolonged hospitalization and that length of stay is influenced by institutional discharge practice, etiology, local complications, and social factors; the composite is therefore not presented as a surrogate for revised Atlanta severity. A sensitivity analysis using the harder endpoint of ICU admission or death was performed, and component-specific results are reported with confidence intervals.

### 2.6. Statistical Analysis

Analyses used IBM SPSS Statistics 26.0 (IBM Corp., Armonk, NY, USA) and Python 3.13.1 (Python Software Foundation, Wilmington, DE, USA); a two-sided *p* < 0.05 was significant. Continuous variables (non-normal by the Shapiro–Wilk test) are given as median (interquartile range, IQR) and compared with the Mann–Whitney U test, and categorical variables as number (percentage) with the chi-squared or Fisher exact test.

For each index the area under the receiver operating characteristic curve (AUC) was estimated with 95% confidence intervals (CI) from DeLong variance [28]; the Youden-optimal cut-off [29] and operating characteristics (Wilson CI) are given in Supplementary Table S4. The eight unadjusted AUCs were compared pairwise (DeLong test, 28 comparisons) with Holm correction. Each index was natural logarithm-transformed, standardized, and entered separately into age-adjusted logistic regression, with effect sizes as adjusted odds ratios (OR) per one-standard-deviation (SD) increase; because adjustment was for age only, indices are described as associated with the outcome after adjustment for age. Model fit, discrimination, and calibration were summarized by the Akaike information criterion (AIC), the bootstrap optimism-corrected AUC (500 resamples), and the internally validated calibration slope and Brier score.

Three focused analyses were pre-specified: (i) incremental value over the standard five-component BISAP score (BISAP alone vs. BISAP plus each index; change in AUC with the DeLong test and the likelihood-ratio test); (ii) comparison of GINI with its components, since GINI equals CAR × PIV (age plus GINI vs. age plus CAR plus PIV); and (iii) an exploratory bedside score with decision-curve analysis [30], detailed with the BISAP definition in the Supplementary Methods. Sensitivity analyses comprised all qualifying admissions with patient-clustered generalized estimating equations (GEE), exclusion of diagnosis-suspect admissions, the inclusive ICU definition, and each outcome component. The 73 events were adequate for the parsimonious two-predictor models but limited precision for comparisons between closely performing indices and the bedside score, which are interpreted as exploratory. BISAP was reconstructed retrospectively from the admission record rather than recorded prospectively: blood urea nitrogen above 25 mg/dL from the admission panel, age above 60 years from demographics, pleural effusion from the structured radiology report, systemic inflammatory response syndrome from admission vital signs together with the white-cell count, and impaired mental status from the clinical narrative. Criteria without available source data were scored as absent, and the number of assessable criteria was recorded for every patient (five of five in 63.9%, four of five in 34.9%, and three of five in 1.3% of the analytic cohort). Because respiratory rate was almost never documented and mental status was frequently unrecorded, BISAP is expected to be mildly underestimated here; incremental value is therefore reported as performance beyond a retrospectively reconstructed BISAP score rather than beyond established severity assessment in general. Five additional analyses were performed: comparison of each composite index with its simplest constituent analytes entered alone; the harder ICU-or-death endpoint; evaluation of every index within its own largest available cohort; analyses stratified by documented vs. undefined etiology with a formal interaction test; and a likelihood-ratio test of the equal-weight constraint that GINI imposes on its components. Calibration slope, calibration intercept, and the Brier score are reported with bootstrap percentile confidence intervals.

## 3. Results

### 3.1. Baseline Characteristics and Outcome Composition

In total, 238 patients were analyzed; the median age was 53 years (IQR 41 to 66), 130 (54.6%) were male, and the median length of stay was 5 days (IQR 3 to 7). The composite outcome occurred in 73 patients (30.7%): 58 had prolonged stay only, nine ICU only, four prolonged stay and ICU, one ICU and death, and one had all three (63 prolonged stay, 15 ICU, two deaths), driven predominantly by prolonged hospitalization. Compared with the 132 excluded patients, the analyzed patients were younger (53 vs. 59 years; *p* = 0.028), more often male (54.6% vs. 41.7%; *p* = 0.023), and admitted more recently, with similar outcome frequency (30.7% vs. 28.0%; *p* = 0.679); full comparisons are in Supplementary Tables S1 and S2. Patients meeting the outcome had higher inflammatory activity, lower immunonutritional reserve, and higher BISAP scores, whereas age and sex did not differ significantly (Table 1). Etiology was undefined in 142 patients (59.7%; biliary 65, alcohol-related 19, hypertriglyceridemic 11, drug-related 1). All eight indices differed significantly between groups (all *p* < 0.001).

### 3.2. Discrimination of Single Indices

All eight indices significantly discriminated the composite outcome, with modest and broadly similar unadjusted AUCs (0.687 to 0.729) and overlapping confidence intervals; CALLY (0.729) and NLR (0.727) were numerically highest, followed by GINI (0.716). At the data-derived Youden thresholds, sensitivity ranged from approximately 47% to 78%, and the negative predictive values largely reflected the local event prevalence of 30.7%; these operating characteristics are insufficient to define a safe rule-out threshold, and we do not propose the indices for that purpose (Table 2; Supplementary Table S4).

### 3.3. Pairwise Comparison of Unadjusted AUCs

Pairwise DeLong testing of the unadjusted AUCs showed no significant difference between any pair after Holm correction (28 comparisons). Two contrasts reached nominal, uncorrected significance, both involving CALLY vs. a CRP-based index (CALLY vs. CCR, *p* = 0.028; CALLY vs. CAR, *p* = 0.040), but neither survived correction. The age-adjusted predicted probabilities gave the same conclusion (smallest raw *p* = 0.043; none significant after Holm). This absence of a significant difference is not evidence of equivalence, given limited power to detect small differences. The complete unadjusted matrix is in Table 3.

### 3.4. Age-Adjusted Analysis, Calibration, and Incremental Value over BISAP

After adjustment for age, all eight indices were associated with the composite outcome (all *p* < 0.001), with standardized adjusted odds ratios per standard deviation from 0.42 to 2.46 (Table 4). Three indices produced the numerically highest, closely similar discrimination with overlapping confidence intervals: NLR (age-adjusted AUC 0.737, 95% CI 0.667 to 0.808), CALLY (0.735, 0.664 to 0.806), and GINI (0.734, 0.661 to 0.806). Bootstrap optimism was small (corrected AUCs 0.728 to 0.729), and the models were well calibrated (calibration slope 0.97 to 1.00; Brier 0.18; Supplementary Table S6a). The ROC curves are shown in Figure 2 and the odds ratios in Figure 3. Calibration of the age-plus-index models was close to ideal, and bootstrap percentile confidence intervals were: calibration slope 1.00 (95% CI 0.66 to 1.54) with intercept 0.00 (−0.335 to 0.464) for NLR, 1.00 (0.65 to 1.48) with 0.00 (−0.319 to 0.392) for CALLY, and 1.00 (0.67 to 1.5) with 0.00 (−0.339 to 0.499) for GINI; Brier scores were 0.177, 0.18, and 0.178 respectively (Supplementary Table S6b). The wide slope intervals show that calibration is estimated imprecisely in a cohort of this size.

Each index added discrimination beyond the retrospectively reconstructed BISAP score. BISAP alone discriminated only modestly (AUC 0.631, 95% CI 0.559 to 0.704). Adding a single index raised the AUC to 0.752 (95% CI 0.681 to 0.822) for CALLY, 0.746 (0.676 to 0.817) for NLR, and 0.738 (0.666 to 0.809) for GINI, with optimism-corrected gains of +0.120, +0.112, and +0.103. All eight indices contributed significantly (likelihood-ratio *p* < 0.001 for each; optimism-corrected change in AUC +0.070 to +0.120; as seen in Table 5 and Supplementary Table S13). Because age is one of the five BISAP components, age was not entered separately. As BISAP was reconstructed retrospectively and is probably underestimated here (only nine patients scored at least 3), this comparison should not be read as superiority over a prospectively applied severity score.

### 3.5. Inter-Index Redundancy

Because the indices share input variables, their correlation structure was examined (Supplementary Figure S1). Spearman correlations confirmed a tightly correlated CRP/albumin cluster (CAR–CCR rho = +1.00; CAR–CALLY rho = −0.96). GINI correlated strongly but not perfectly with each component (GINI–CAR rho = +0.89; GINI–PIV rho = +0.80) and with NLR (rho = +0.74). Overall, the eight indices captured a few correlated host-response signals rather than eight independent ones. Given this redundancy, we asked whether the composite indices outperform their simplest constituents. Entered alone and adjusted for age, lymphocyte count reached an AUC of 0.703 (95% CI 0.629 to 0.778), CRP 0.702 (0.628 to 0.776), neutrophil count 0.683, calcium 0.650, and albumin 0.623 (0.544 to 0.702). CALLY discriminated significantly better than CRP alone (*p* = 0.014) and better than albumin alone (*p* = 0.012), and NLR and GINI exceeded albumin (*p* = 0.025 and *p* = 0.020); neither NLR nor GINI improved significantly on CRP alone (*p* = 0.331 and *p* = 0.130). The incremental benefit of compositing is therefore modest and is clearest against albumin, not against CRP (Supplementary Table S9).

### 3.6. GINI vs. Its Components

Because GINI equals CAR × PIV, we compared it with its constituents. GINI showed numerically higher discrimination than either component (age-adjusted AUC 0.734 vs. 0.710 for CAR and 0.708 for PIV) and a larger standardized effect (adjusted OR 2.43 vs. 2.04 and 2.17), but these differences were not significant. Against a model with both components entered separately (age plus CAR plus PIV, AUC 0.729), the single-term GINI model (0.734) performed comparably (DeLong *p* = 0.541) with a marginally lower AIC (264.0 vs. 265.2). GINI thus behaves as a parsimonious summary of its two components rather than a source of added discrimination; its apparent advantage over CAR and PIV is numerical, not statistically demonstrated. Because a single GINI term constrains the log-components to equal weight, we formally tested that constraint: the unrestricted coefficients differed numerically (log CAR 0.302, log PIV 0.499), but the likelihood-ratio test did not reject the constraint (chi-square 0.75, 1 df, *p* = 0.387) and the constrained model had a marginally lower AIC (264.0 vs. 265.2). GINI is therefore an acceptable parsimonious summary of CAR and PIV rather than a source of additional discrimination; the absence of a significant difference does not establish equivalence.

### 3.7. Exploratory Bedside Score and Decision-Curve Analysis

To illustrate bedside translation, an integer score combining age category with the CALLY tertile, and a decision-curve analysis, were derived. Because these steps (selecting CALLY, tertile estimation, and score construction) were performed in the same cohort in which they were evaluated, they are exploratory and reported in full in the Supplementary Materials (Supplementary Table S8 and Supplementary Figures S2 and S3). Briefly, the score stratified observed risk from 12.9% to 62.5%, and net benefit was concentrated at low-to-moderate thresholds. These analyses are hypothesis-generating and not proposed for clinical use.

### 3.8. Sensitivity Analyses

The findings were robust across sensitivity analyses. The exclusion of the nine diagnosis-suspect admissions (seven with the outcome) preserved the ranking and slightly increased the leading AUCs (CALLY 0.753, GINI 0.747, NLR 0.741; Supplementary Table S3). In the patient-clustered analysis of all qualifying admissions with a complete panel (335 admissions; one with PIV = 0 excluded), the leading indices retained strong associations (adjusted OR per SD: NLR 2.25, CALLY 0.42, GINI 2.45; all *p* < 0.001; Supplementary Table S7). The inclusive ICU criterion (25 ICU admissions; 77 composite events, 32.4%) did not change the results (Supplementary Note S1). By component (Supplementary Table S5), NLR discriminated ICU admission best (AUC 0.797) and CALLY prolonged stay best (0.700); the death component (two events) was too infrequent for separate analysis. Three additional analyses are of particular note. First, for the harder endpoint of ICU admission or death (15 events), discrimination was consistently higher than for the composite: NLR 0.828 (95% CI 0.725 to 0.930), CALLY 0.816 (0.713 to 0.919), PNI 0.803, and GINI 0.779, with all eight indices remaining significantly associated; confidence intervals are wide and these estimates are exploratory, but they indicate that the indices track clinically severe events at least as well as the length of stay-dominated composite (Supplementary Table S10). Second, when each index was evaluated in its own largest available cohort rather than the common complete-case cohort, discrimination was essentially unchanged (NLR, *n* = 325, AUC 0.719; CALLY, *n* = 242, AUC 0.742; CCR, *n* = 298, AUC 0.702), arguing against major distortion from complete-case selection (Supplementary Table S11). Third, in analyses stratified by etiology, discrimination was numerically higher among patients with undefined etiology (*n* = 142) than among those with a documented cause (*n* = 96); the interaction was not significant for NLR (*p* = 0.119) or CALLY (*p* = 0.243) but reached nominal significance for GINI (*p* = 0.039), reported as hypothesis-generating given multiple testing (Supplementary Table S12).

## 4. Discussion

In this exploratory single-center cohort of 238 patients with AP, eight admission indices were compared for their association with a composite adverse in-hospital outcome (death, ICU admission, or prolonged hospitalization). After age adjustment, all eight were associated with the outcome and retained modest, optimism-corrected, well-calibrated discrimination. Three indices were numerically highest, with no significant pairwise difference after correction: NLR, CALLY, and GINI, each adding discrimination beyond BISAP. GINI (= CAR × PIV) showed numerically higher but not significant discrimination than its components. An exploratory bedside score and decision-curve analysis are in the Supplementary Materials.

The central finding is similarity rather than superiority. NLR, CALLY, and GINI clustered at the top (age-adjusted AUC 0.734 to 0.737), but their confidence intervals overlapped and no pairwise comparison was significant after correction. This reflects an absence of detectable difference in a modestly sized cohort, not demonstrated equivalence, which would require a pre-specified margin and larger sample. The predictive information carried by these indices is thus largely shared, reflecting a few correlated host-response signals rather than eight independent biomarkers. Practically, the choice among the leading indices can be guided by availability: NLR needs only a complete blood count, whereas CALLY and GINI additionally require CRP and albumin. A related question is whether these composites are worth constructing at all. In our data the CRP/albumin cluster (CAR, CCR, CALLY) behaves as a single CRP-driven signal, and CRP entered alone already achieved an age-adjusted AUC of 0.702. CALLY improved significantly on both CRP and albumin, whereas NLR and GINI improved on albumin but not on CRP. The honest interpretation is that compositing yields a modest and inconsistent gain over the single best analyte, and that a more elaborate index should not be assumed to be a better one.

GINI is not a new index: it was developed by Topkan and colleagues in oncology as the product of CAR and PIV [20]. The contribution here is its first evaluation in AP and direct comparison with its constituents, not its creation. GINI showed numerically higher discrimination than CAR or PIV alone, but not significantly, and a model containing GINI performed comparably to one containing both components separately (DeLong *p* = 0.541) with a marginally lower AIC. GINI therefore behaves as a parsimonious summary of a CRP/albumin signal (CAR) and a cellular immune-inflammatory signal (PIV), not a source of added discrimination. Given this and its equal footing with the simpler, more available NLR, GINI is best regarded as one reasonable option among several correlated AP indices.

Individual index performance was broadly consistent with prior AP reports. NLR is the most studied, with a meta-analysis confirming its association with severity [21,22]; its strong performance, especially for the ICU component, fits its role as a systemic inflammation marker. CAR has shown AUCs of about 0.65 to 0.75 [14,15] and was recently the strongest predictor of prolonged hospitalization in AP [24]. CALLY was recently associated with progression to severe AP [23]. Evidence for HALP is more limited [25]. The near-perfect collinearity among the CRP-dominated indices (CAR, CCR, CALLY) follows from their shared CRP-dominated construction, so they behave as largely redundant representatives of one host-response signal.

These results should also be read against the biological and management heterogeneity of pancreatic disease. Albumin- and lymphocyte-based indices are not pure nutritional measures: in pancreatic disease inflammation, malabsorption, and body composition loss interact closely, so a low albumin or lymphocyte count reflects a mixture of inflammatory burden and depleted reserve rather than nutrition alone [31]. Etiological heterogeneity is also substantial, and unsupervised analyses show that cases labeled idiopathic comprise distinct phenotypes with different clinical profiles [32]; this is directly relevant to the 59.7% of our cohort with undefined etiology and may contribute to the stratified differences observed. Finally, both outcome components are management-dependent: local complications such as pseudocysts and walled-off necrosis drive imaging and drainage pathways that lengthen admission [33], and the absence of standardized intervention thresholds in necrotizing pancreatitis means that the timing of escalation and discharge varies between centers [34]. These considerations reinforce our decision to treat the composite as center-dependent rather than as a severity surrogate.

We chose a composite rather than any single endpoint. Mortality alone (two deaths) would be underpowered, whereas length of stay alone would miss ICU escalation and death. The trade-off is that the composite was driven predominantly by prolonged hospitalization (63 of 73 events), influenced by non-biological factors, so the results pertain mainly to prolonged in-hospital course. The most clinically relevant observation is that the leading indices added discrimination beyond BISAP, raising its AUC from 0.63 to about 0.75 (change 0.11 to 0.12; likelihood-ratio *p* < 0.001). Because BISAP is only a partial proxy and etiology and organ failure data were incomplete, this indicates, without proving independence, that these inexpensive indices are not merely restating a routine severity score. The three components are not clinically equivalent, and length of stay is partly administrative; we therefore do not present the composite as a surrogate for revised Atlanta severity. Reassuringly, the ICU-or-death analysis shows that discrimination does not depend on the length-of-stay component: for the harder ICU-or-death endpoint the age-adjusted AUCs were numerically higher (0.73 to 0.83) than for the composite, indicating that the indices capture clinically severe events rather than merely local discharge practice. That analysis rests on only 15 events, so its confidence intervals are wide and it cannot by itself establish performance in severe disease; it does, however, argue against the interpretation that our findings are an artifact of length-of-stay variation.

Any clinical use would most plausibly be admission-based risk triage, in which a high-risk value prompts closer monitoring or earlier senior review rather than a binary escalation decision. Delayed discharge is not proposed as an illustrative action, because prolonged hospitalization is itself the dominant component of the endpoint and such an action would be circular. The exploratory bedside score and decision-curve analysis remain in the Supplementary Materials and are reported only to illustrate potential translation: index selection, cut-points, and evaluation all occurred in the same dataset, so their apparent performance is optimistic and they are not proposed for clinical use.

Several limitations apply. First, the single-center retrospective design limits causal inference and generalizability. Second, complete-case selection was substantial (132 of 370 index admissions, 35.7%, excluded), and excluded patients were older and less often male, so estimates apply to patients with a complete admission panel. Third, exact laboratory timestamps were unavailable, so temporal leakage for the ICU component cannot be fully excluded; the analysis should ideally be repeated with strictly time-anchored, pre-outcome values. Fourth, the composite was dominated by prolonged hospitalization, a partly administrative outcome, and etiology was undefined in most patients. Fifth, adjustment was for age and BISAP only; the full Atlanta classification, organ failure, and scores such as APACHE II were unavailable. Sixth, GINI was derived in oncology. Finally, cut-offs and the ranking were derived in the same sample, so external validation in a prospective multicenter cohort is required before clinical use. Additional limitations should be emphasized. Laboratory acquisition times were not retrievable, so temporal precedence over ICU transfer could not be verified for every patient and reverse influence of the results on clinical decisions cannot be excluded. Complete-case selection removed 35.7% of index admissions; although each index performed similarly in its own largest available cohort, multiple imputation of the derived indices was not attempted and residual selection bias remains possible. Etiology was undefined in 59.7% of patients, limiting adjustment for cause. BISAP was reconstructed retrospectively, with respiratory rate essentially unavailable and mental status frequently unrecorded, so it is probably underestimated and is only a weak severity benchmark. Finally, the ICU-or-death analysis rests on 15 events, the etiology-stratified interaction tests were not corrected for multiplicity, and no external validation was performed.

The clearest next step is a larger, prospective, multicenter study comparing these indices directly with BISAP, APACHE II, and the revised Atlanta classification, using strictly time-anchored admission values and harder endpoints such as persistent organ failure. Formal testing of the incremental value of composite indices over their components, and of serial vs. static measurement, would also help. Because these tests are inexpensive and routine, their real-world impact on escalation and discharge decisions warrants evaluation.

## 5. Conclusions

In adults hospitalized with acute pancreatitis, several admission inflammatory and immunonutritional indices, led by NLR, CALLY, and GINI, showed modest, broadly comparable discrimination (no significant pairwise difference after correction) for a composite adverse in-hospital outcome and added information beyond BISAP. No index was demonstrably superior, GINI performed comparably to its components, and the composite was driven largely by prolonged hospitalization. These exploratory findings require prospective external validation, including direct comparison with established severity assessment, before any clinical application. These indices add information to a retrospectively reconstructed BISAP score and remain associated with the harder ICU-or-death endpoint, but they do not consistently outperform CRP measured alone, and their incremental value over routine clinical assessment remains unproven. The findings are exploratory and require prospective external validation before clinical application.

## Figures and Tables

**Figure 1:**
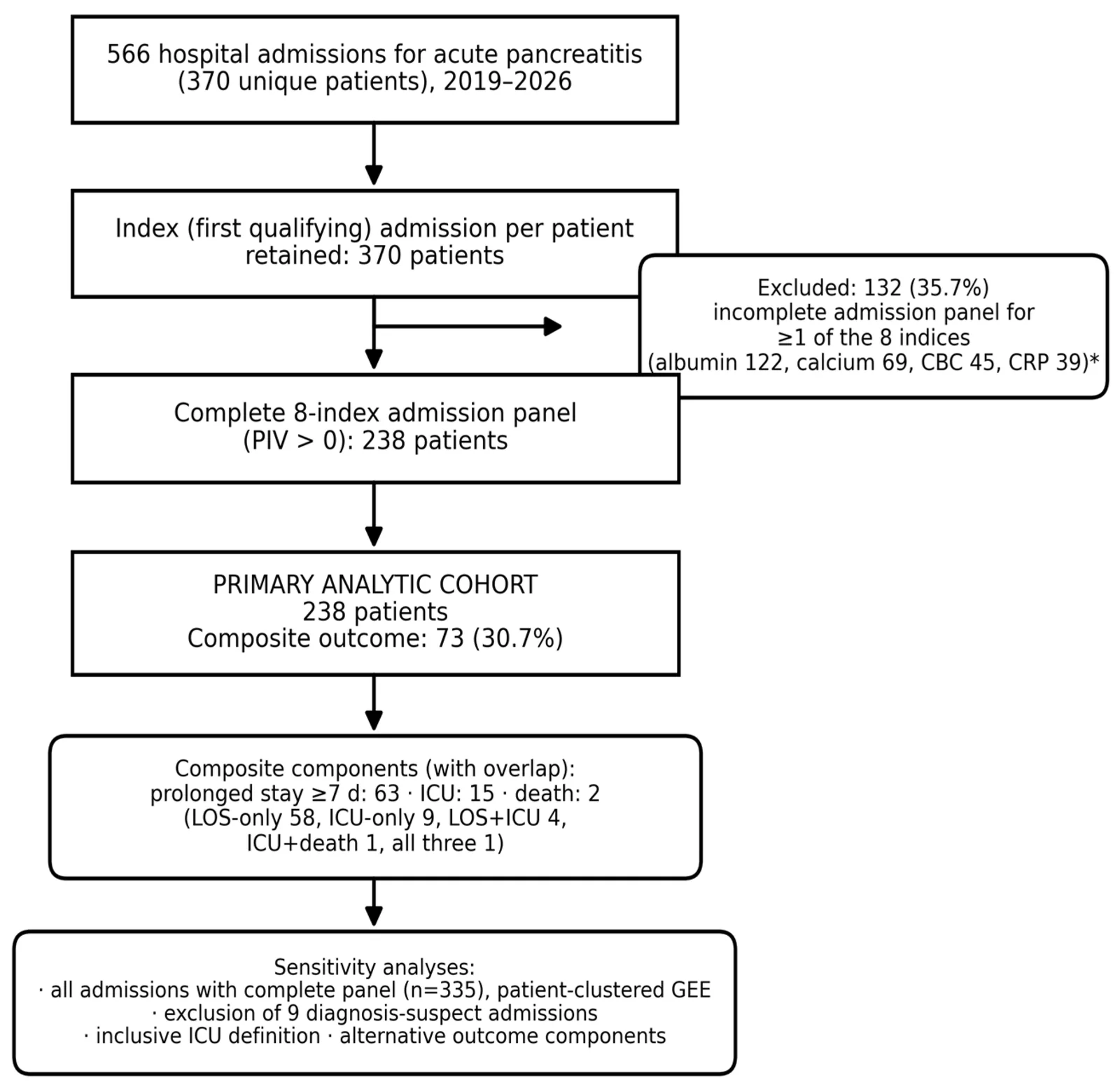
Study flow diagram. * Counts of missing components are not mutually exclusive. GEE, generalized estimating equations; ICU, intensive care unit; LOS, length of stay; PIV, pan-immune-inflammation value.

**Figure 2:**
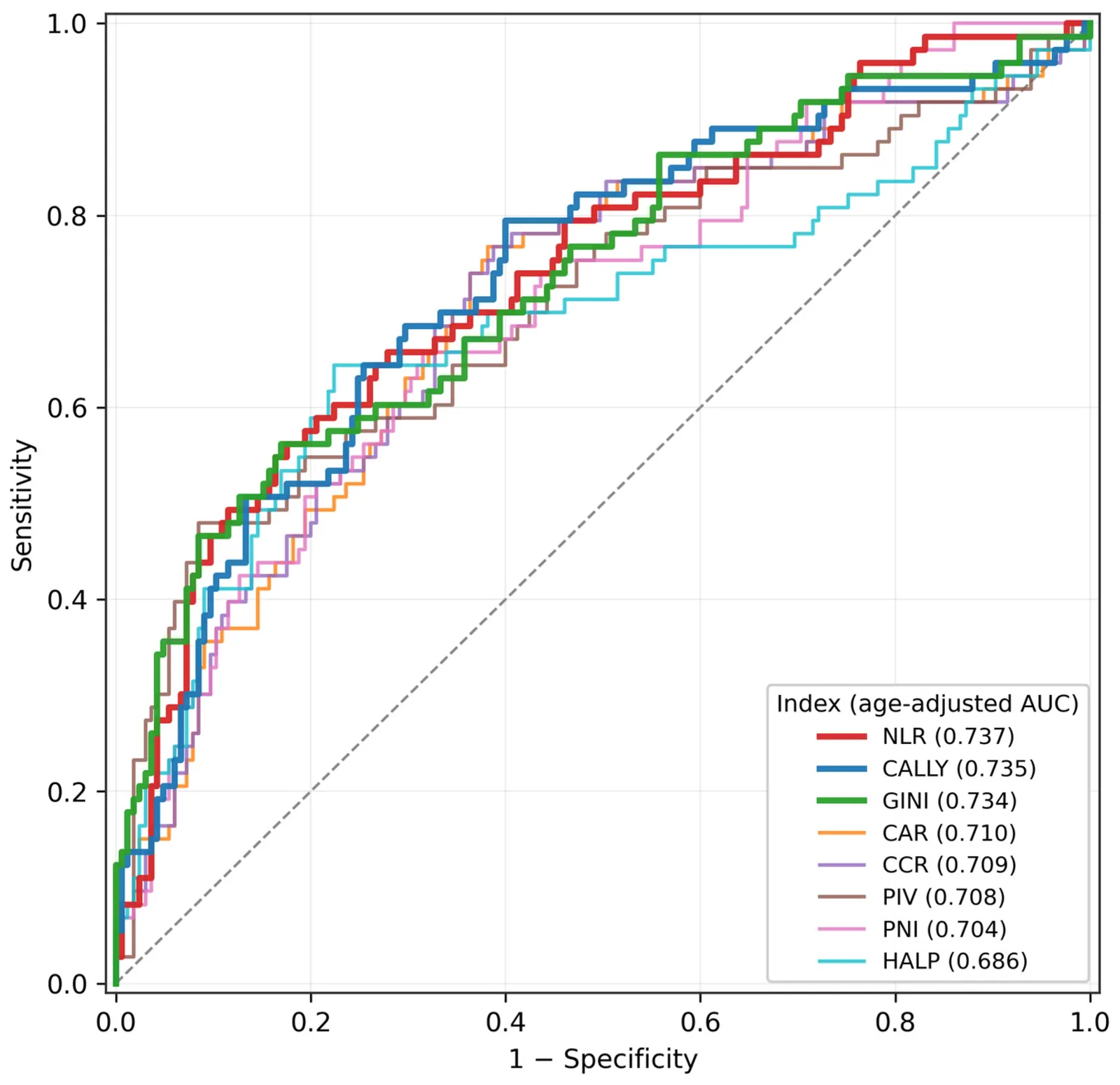
Age-adjusted receiver operating characteristic curves of the eight indices for the composite adverse in-hospital outcome (238 patients). The AUC of each single-index model is given in the legend. The dashed diagonal line indicates no discrimination (AUC = 0.50).

**Figure 3:**
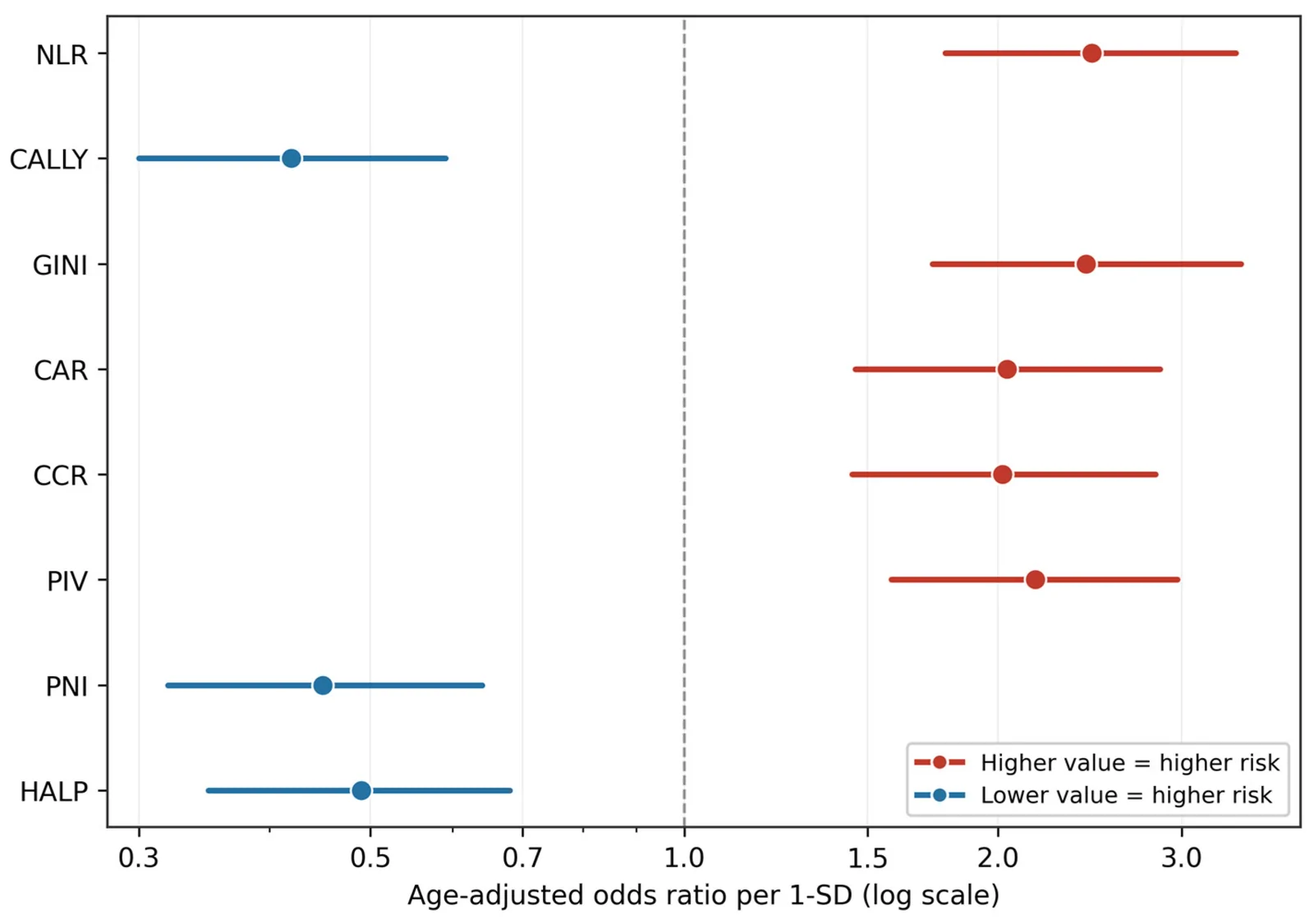
Forest plot of age-adjusted odds ratios per one-standard-deviation change in the natural logarithm-transformed index. Red markers denote indices in which higher values indicate higher risk (NLR, GINI, PIV, CAR, CCR); blue markers denote indices in which lower values indicate higher risk (CALLY, PNI, HALP). Horizontal lines are 95% confidence intervals; the x-axis is on a logarithmic scale; and the dashed vertical line marks the null value (odds ratio = 1.00).

**Table 1:** Baseline characteristics by composite adverse in-hospital outcome.

Variable	All Patients (*n* = 238)	No Event (*n* = 165)	Composite Event (*n* = 73)	*p*
Age, years	53 (41–66)	52 (40–64)	56 (44–70)	0.066
Male sex, *n* (%)	130 (54.6)	93 (56.4)	37 (50.7)	0.503
Length of stay, days	5 (3–7)	4 (3–5)	9 (7–11)	NA
**Clinical severity, etiology, and comorbidity**				
BISAP score	0 (0–1)	0 (0–1)	1 (0–1)	<0.001
BISAP ≥ 3, *n* (%)	9 (3.8%)	3 (1.8%)	6 (8.2%)	0.026
Necrotizing pancreatitis, *n* (%)	11 (4.6%)	3 (1.8%)	8 (11.0%)	0.006
Biliary etiology, *n* (%)	65 (27.3%)	40 (24.2%)	25 (34.2%)	0.150
Comorbidity count	1 (0–2)	1 (0–2)	1 (0–2)	0.109
Diabetes mellitus, *n* (%)	106 (44.5%)	70 (42.4%)	36 (49.3%)	0.398
Hypertension, *n* (%)	72 (30.3%)	46 (27.9%)	26 (35.6%)	0.296
**Admission laboratory values**				
WBC, ×10^3^/μL	9.25 (7.21–12.32)	8.87 (7.09–10.78)	11.08 (7.59–15.93)	0.002
Neutrophils, ×10^3^/μL	6.75 (4.76–9.89)	6.36 (4.56–8.77)	9.34 (5.47–13.85)	<0.001
Lymphocytes, ×10^3^/μL	1.54 (1.04–2.07)	1.69 (1.26–2.19)	1.07 (0.80–1.66)	<0.001
Monocytes, ×10^3^/μL	0.54 (0.40–0.74)	0.50 (0.40–0.68)	0.58 (0.42–0.86)	0.053
Hemoglobin, g/L	124 (107–139)	124 (109–139)	121 (104–142)	0.655
Platelets, ×10^3^/μL	207 (165–253)	207 (165–248)	218 (164–279)	0.373
Amylase, U/L	244 (97–548)	230 (97–458)	376 (94–682)	0.123
Lipase, U/L	265 (91–781)	251 (88–653)	286 (95–1407)	0.167
AST, U/L	31 (19–88)	27 (18–73)	42 (25–130)	0.006
ALT, U/L	34 (17–135)	30 (16–103)	47 (18–188)	0.048
ALP, U/L	88 (66–132)	81 (63–119)	120 (75–177)	0.001
LDH, U/L	180 (147–235)	165 (140–208)	224 (179–385)	<0.001
BUN, mg/dL	10.9 (8.0–16.2)	10.0 (8.0–15.0)	12.0 (8.0–21.7)	0.038
Creatinine, mg/dL	0.75 (0.60–0.93)	0.74 (0.61–0.88)	0.75 (0.58–1.02)	0.376
Glucose, mg/dL	105 (89–138)	102 (89–130)	122 (88–163)	0.033
CRP, mg/L	42.4 (12.3–114.7)	29.0 (10.0–81.1)	89.7 (39.3–189.3)	<0.001
Albumin, g/L	36 (33–39)	36 (34–39)	35 (32–38)	0.002
Calcium, mg/dL	8.69 (8.27–9.01)	8.74 (8.44–9.10)	8.53 (7.89–8.85)	0.001
**Indices**				
NLR	4.17 (2.47–9.29)	3.55 (2.27–5.95)	9.16 (4.07–18.29)	<0.001
CAR	1.15 (0.37–3.30)	0.81 (0.26–2.21)	2.80 (1.11–5.32)	<0.001
CCR	5.09 (1.47–13.56)	3.35 (1.12–8.93)	11.60 (4.88–20.92)	<0.001
PIV	475 (250–1023)	422 (219–778)	974 (353–2713)	<0.001
GINI	461 (119–2732)	313 (93–1312)	2019 (224–10,489)	<0.001
PNI	44.2 (39.9–47.4)	44.8 (41.5–48.8)	40.7 (36.4–44.6)	<0.001
HALP	33.5 (20.2–46.2)	37.1 (26.1–47.0)	22.0 (12.6–37.5)	<0.001
CALLY	0.13 (0.04–0.53)	0.19 (0.07–0.62)	0.04 (0.02–0.17)	<0.001

Values are median (IQR) unless otherwise specified. Abbreviations: ALP, alkaline phosphatase; ALT, alanine aminotransferase; AST, aspartate aminotransferase; BISAP, Bedside Index for Severity in Acute Pancreatitis; BUN, blood urea nitrogen; CALLY, CRP-albumin-lymphocyte index; CAR, CRP-to-albumin ratio; CCR, CRP-to-calcium ratio; CRP, C-reactive protein; GINI, Global Immune-Nutrition-Inflammation Index; HALP, hemoglobin-albumin-lymphocyte-platelet score; NLR, neutrophil-to-lymphocyte ratio; PIV, pan-immune-inflammation value; PNI, prognostic nutritional index; IQR, interquartile range; LDH, lactate dehydrogenase; NA, not applicable; WBC, white blood cell count.

**Table 2:** Unadjusted discrimination of the eight indices for the composite adverse in-hospital outcome.

Index	AUC (95% CI)	Cut-Off	Direction	Sensitivity, %	Specificity, %	PPV, %	NPV, %
NLR	0.727 (0.653–0.801)	6.33	>	65.8	76.4	55.2	83.4
CALLY	0.729 (0.656–0.802)	0.05	≤	60.3	83.6	62.0	82.6
GINI	0.716 (0.641–0.792)	4082.32	>	46.6	89.7	66.7	79.1
PNI	0.705 (0.633–0.778)	41.95	≤	61.6	72.1	49.5	81.0
CAR	0.702 (0.626–0.778)	2.46	>	56.2	78.8	53.9	80.2
CCR	0.700 (0.625–0.776)	4.55	>	78.1	58.2	45.2	85.7
HALP	0.689 (0.607–0.770)	25.44	≤	61.6	76.4	53.6	81.8
PIV	0.687 (0.605–0.768)	871.49	>	57.5	80.6	56.8	81.1

Cut-offs were determined by maximizing the Youden index. The “Direction” column indicates whether values above (>) or at or below (≤) the cut-off denote higher risk. Abbreviations: AUC, area under the receiver operating characteristic curve; CALLY, CRP-albumin-lymphocyte index; CAR, CRP-to-albumin ratio; CCR, CRP-to-calcium ratio; GINI, Global Immune-Nutrition-Inflammation Index; HALP, hemoglobin-albumin-lymphocyte-platelet score; NLR, neutrophil-to-lymphocyte ratio; PIV, pan-immune-inflammation value; PNI, prognostic nutritional index; CI, confidence interval; NPV, negative predictive value; PPV, positive predictive value.

**Table 3:** DeLong pairwise comparison of the unadjusted single-index AUCs.

	NLR	CALLY	GINI	CAR	CCR	PIV	PNI	HALP
NLR	–	0.953	0.717	0.528	0.491	0.155	0.576	0.329
CALLY	0.953	–	0.539	0.040	0.028	0.326	0.529	0.367
GINI	0.717	0.539	–	0.510	0.451	0.288	0.799	0.526
CAR	0.528	0.040	0.510	–	0.709	0.741	0.939	0.793
CCR	0.491	0.028	0.451	0.709	–	0.766	0.911	0.821
PIV	0.155	0.326	0.288	0.741	0.766	–	0.711	0.967
PNI	0.576	0.529	0.799	0.939	0.911	0.711	–	0.586
HALP	0.329	0.367	0.526	0.793	0.821	0.967	0.586	–

Values are raw (uncorrected) two-sided *p*-values from the DeLong test for two correlated AUCs. No comparison remained significant after Holm correction. Abbreviations: AUC, area under the receiver operating characteristic curve; CALLY, CRP-albumin-lymphocyte index; CAR, CRP-to-albumin ratio; CCR, CRP-to-calcium ratio; GINI, Global Immune-Nutrition-Inflammation Index; HALP, hemoglobin-albumin-lymphocyte-platelet score; NLR, neutrophil-to-lymphocyte ratio; PIV, pan-immune-inflammation value; PNI, prognostic nutritional index.

**Table 4:** Age-adjusted logistic regression for the log-transformed indices.

Index	Adjusted OR per 1-SD (95% CI)	*p*	AUC (95% CI)	Bootstrap-Corrected AUC	AIC
NLR	2.46 (1.78–3.38)	<0.001	0.737 (0.667–0.808)	0.729	260.3
CALLY	0.42 (0.30–0.59)	<0.001	0.735 (0.664–0.806)	0.729	266.0
GINI	2.43 (1.73–3.42)	<0.001	0.734 (0.661–0.806)	0.728	264.0
CAR	2.04 (1.46–2.86)	<0.001	0.710 (0.636–0.784)	0.701	275.3
CCR	2.02 (1.45–2.83)	<0.001	0.709 (0.635–0.783)	0.700	275.8
PIV	2.17 (1.58–2.97)	<0.001	0.708 (0.631–0.786)	0.699	269.4
PNI	0.45 (0.32–0.64)	<0.001	0.704 (0.632–0.776)	0.697	271.3
HALP	0.49 (0.35–0.68)	<0.001	0.686 (0.605–0.768)	0.678	274.6

All models are adjusted for age. AUC shown with 95% DeLong confidence interval. Bootstrap-corrected AUC and calibration were obtained over 500 resamples. Abbreviations: AIC, Akaike information criterion; AUC, area under the receiver operating characteristic curve; CALLY, CRP-albumin-lymphocyte index; CAR, CRP-to-albumin ratio; CCR, CRP-to-calcium ratio; GINI, Global Immune-Nutrition-Inflammation Index; HALP, hemoglobin-albumin-lymphocyte-platelet score; NLR, neutrophil-to-lymphocyte ratio; PIV, pan-immune-inflammation value; PNI, prognostic nutritional index; CI, confidence interval; OR, odds ratio; SD, standard deviation.

**Table 5:** Incremental discrimination of the three leading indices beyond BISAP.

Model	AUC (95% CI)	ΔAUC vs. Base	DeLong *p*	LRT *p*
BISAP alone	0.631 (0.559–0.704)	NA	NA	NA
BISAP + NLR	0.746 (0.676–0.817)	+0.115	0.004	<0.001
BISAP + CALLY	0.752 (0.681–0.822)	+0.121	0.001	<0.001
BISAP + GINI	0.738 (0.666–0.809)	+0.107	0.004	<0.001

Base model: BISAP alone (AUC 0.631). Because age is a BISAP component, age is not entered separately. Abbreviations: AUC, area under the receiver operating characteristic curve; BISAP, Bedside Index for Severity in Acute Pancreatitis; CALLY, CRP-albumin-lymphocyte index; CI, confidence interval; ΔAUC, change in AUC vs. the base model; DeLong *p*, significance of the AUC change; GINI, Global Immune-Nutrition-Inflammation Index; LRT *p*, likelihood-ratio test for adding the index; NA, not applicable; NLR, neutrophil-to-lymphocyte ratio.

## Data Availability

The data supporting this study are available on reasonable request from the corresponding author, subject to institutional restrictions.

## Supplementary Materials

The following supporting information can be downloaded at: https://www.mdpi.com/article/10.3390/jcm15197495/s1, Supplementary Methods (BISAP score, exploratory bedside score, decision-curve analysis); Table S1: Missingness of each index component among the 370 index admissions; Table S2: Comparison of included and excluded index admissions; Table S3: Sensitivity analysis excluding diagnosis-suspect admissions; Table S4: Operating characteristics of each index at the Youden cut-off, with 95% Wilson confidence intervals; Table S5: Discrimination (unadjusted AUC) of each index for the composite outcome and its components; Table S6a: Internally validated calibration of the three leading age-adjusted models; Table S6b: Internally validated calibration with 95% confidence intervals; Table S7: Patient-clustered (GEE) association of the three leading indices with the composite outcome; Table S8: Distribution and observed composite-outcome rate by the exploratory bedside integer score; Table S9: Discrimination of the simplest constituent analytes entered alone, compared with the composite indices; Table S10: Sensitivity analysis using the harder endpoint of ICU admission or death (15 events); Table S11: Performance of each index within its own largest available cohort; Table S12: Discrimination stratified by documented versus undefined etiology; Table S13: Incremental discrimination of all eight indices beyond the retrospectively reconstructed BISAP score; Supplementary Methods (addendum): Retrospective reconstruction of the BISAP score. Note S1: Sensitivity analysis using an inclusive ICU definition; Figure S1: Spearman correlation matrix of the eight log-transformed indices; Figure S2: Decision-curve analysis of the three leading age-adjusted models (exploratory); Figure S3: Observed composite-outcome rate by the exploratory bedside integer score (0 to 4).
