# Supplementary material for: Comparative Prognostic Performance of Inflammatory and Immunonutritional Indices in Acute Pancreatitis: A Head-to-Head Cohort Study
Source: J Clin Med. 2026 Sep 26;15(19):7495. doi: 10.3390/jcm15197495 (PMC13666038; doi:10.3390/jcm15197495)
Supplement: Supplementary file 1 [file jcm-15-07495-s001.zip › jcm-4514213-supplementary.pdf]

Supplementary Material

Supplementary Methods

BISAP score. Incremental value over established severity assessment was tested against the standard five-component Bedside Index for Severity in Acute Pancreatitis (BISAP): blood urea nitrogen > 25 mg/dL, impaired mental status, systemic inflammatory response syndrome, age > 60 years, and pleural effusion (range 0 to 5). The score was reconstructed from the admission record (observed range 0 to 4 in this cohort). BISAP alone was compared with BISAP plus each index using the change in AUC (with 95% confidence intervals and the DeLong test) and the likelihood-ratio test. Because age is already one of the five BISAP components, age was not entered separately; models additionally containing a separate age term were examined only as a secondary check and did not change the conclusions.

Exploratory bedside score. An integer score combined an age category (0 for age < 65 years, 1 for 65 to 74 years, 2 for at least 75 years) with the tertile of a representative index (0 for the lowest-risk to 2 for the highest-risk tertile); CALLY was used because it produced the most monotonic gradient among the leading indices. The exact CALLY tertile thresholds (0.0555 and 0.3018 in this cohort) are reported in Table S8. Internal validation re-estimated the tertile thresholds within each bootstrap sample, so that the reported optimism reflects the data-driven cut-points; because the prior selection of CALLY among the leading indices is not itself repeated in the bootstrap, the true optimism may be slightly larger, and the score is presented as exploratory only.

Decision-curve analysis. Clinical utility was explored by decision-curve analysis, in which classification as high risk was linked to a plausible action (closer monitoring or earlier senior review). Because the curves were derived and evaluated in the same cohort, they are presented as exploratory **(Figure S2)**.

Table S1. Missingness of each index component among the 370 index admissions.

Component	Missing, n	Missing, %
CRP	39	10.5%
Albumin	122	33.0%
Calcium	69	18.6%
Neutrophil	45	12.2%
Lymphocyte	45	12.2%
Monocyte	45	12.2%
Hemoglobin	45	12.2%
Platelet	45	12.2%

Denominator = 370 index admissions. Counts are not mutually exclusive (an admission may be missing several components). Complete-case analysis required all eight indices and therefore excluded 132 admissions (35.7%).

Table S2. Comparison of included and excluded index admissions.

Characteristic	Included (n = 238)	Excluded (n = 132)	p
Age, years, median (IQR)	53 (41–66)	59 (46–69)	0.028
Male sex, n (%)	130 (54.6%)	55 (41.7%)	0.023
Composite outcome, n (%)	73 (30.7%)	37 (28.0%)	0.679
Admission year, median (IQR)	2024 (2023–2025)	2022 (2021–2024)	<0.001

Excluded patients (incomplete eight-index panel) were older, less often male, and admitted in earlier years, although the composite-outcome frequency was similar. In an age-adjusted analysis that additionally included admission year, the associations of the leading indices were essentially unchanged (adjusted OR per 1-SD: NLR 2.46, CALLY 0.42, GINI 2.43; all $p < 0.001$), indicating that calendar-time selection did not materially bias the index associations. Continuous variables compared with the Mann-Whitney U test, categorical with the chi-squared test. Abbreviations: CALLY, CRP-albumin-lymphocyte index; GINI, Global Immune-Nutrition-Inflammation Index; NLR, neutrophil-to-lymphocyte ratio; IQR, interquartile range; OR, odds ratio; SD, standard deviation.

Table S3. Sensitivity analysis excluding diagnosis-suspect admissions.

Index	Age-adjusted AUC, primary cohort (n = 238)	Excluding suspects (n = 229)
NLR	0.737	0.741
CALLY	0.735	0.753
GINI	0.734	0.747

Nine diagnosis-suspect admissions were present in the primary cohort, of which seven met the composite outcome. Their exclusion preserved the ranking and slightly increased the age-adjusted AUCs of the leading indices. Abbreviations: AUC, area under the receiver operating characteristic curve; CALLY, CRP-albumin-lymphocyte index; GINI, Global Immune-Nutrition-Inflammation Index; NLR, neutrophil-to-lymphocyte ratio.

Table S4. Operating characteristics of each index at the Youden cut-off, with 95% Wilson confidence intervals.

Index	Sensitivity, % (95% CI)	Specificity, % (95% CI)	PPV, % (95% CI)	NPV, % (95% CI)
NLR	65.8 (54.3–75.6)	76.4 (69.3–82.2)	55.2 (44.7–65.2)	83.4 (76.7–88.5)
CALLY	60.3 (48.8–70.7)	83.6 (77.2–88.5)	62 (50.3–72.4)	82.6 (76.2–87.6)
GINI	46.6 (35.6–57.9)	89.7 (84.1–93.5)	66.7 (53–78)	79.1 (72.8–84.4)
CAR	56.2 (44.8–67)	78.8 (71.9–84.3)	53.9 (42.8–64.7)	80.2 (73.4–85.6)
CCR	78.1 (67.3–86)	58.2 (50.6–65.4)	45.2 (36.8–53.9)	85.7 (78–91)
PIV	57.5 (46.1–68.2)	80.6 (73.9–85.9)	56.8 (45.4–67.4)	81.1 (74.4–86.4)
PNI	61.6 (50.2–71.9)	72.1 (64.8–78.4)	49.5 (39.4–59.5)	81 (73.8–86.5)
HALP	61.6 (50.2–71.9)	76.4 (69.3–82.2)	53.6 (43–63.8)	81.8 (75–87.1)

PPV and NPV depend on the local composite-outcome prevalence of 30.7%. Abbreviations: CALLY, CRP-albumin-lymphocyte index; CAR, CRP-to-albumin ratio; CCR, CRP-to-calcium ratio; GINI, Global Immune-Nutrition-Inflammation Index; HALP, hemoglobin-albumin-lymphocyte-platelet score; NLR, neutrophil-to-lymphocyte ratio;

PIV, pan-immune-inflammation value; PNI, prognostic nutritional index; CI, confidence interval; NPV, negative predictive value; PPV, positive predictive value.

Table S5. Discrimination (unadjusted AUC) of each index for the composite outcome and its components.

Index	Composite	Prolonged stay (LOS ≥ 7 d)	ICU admission	In-hospital death
NLR	0.727	0.681	0.797	NA
CALLY	0.729	0.700	0.765	NA
GINI	0.716	0.692	0.718	NA
CAR	0.702	0.681	0.736	NA
CCR	0.700	0.679	0.727	NA
PIV	0.687	0.654	0.687	NA
PNI	0.705	0.661	0.786	NA
HALP	0.689	0.666	0.707	NA

Unadjusted AUCs. The in-hospital death component (2 events) was too infrequent for reliable estimation (NA). Abbreviations: AUC, area under the receiver operating characteristic curve; CALLY, CRP-albumin-lymphocyte index; CAR, CRP-to-albumin ratio; CCR, CRP-to-calcium ratio; GINI, Global Immune-Nutrition-Inflammation Index; HALP, hemoglobin-albumin-lymphocyte-platelet score; NLR, neutrophil-to-lymphocyte ratio; PIV, pan-immune-inflammation value; PNI, prognostic nutritional index; ICU, intensive care unit; LOS, length of stay.

Table S6a. Internally validated calibration of the three leading age-adjusted models.

Model	Bootstrap-corrected calibration slope	Bootstrap-corrected Brier score
Age + NLR	0.97	0.182
Age + CALLY	0.99	0.185
Age + GINI	1.00	0.183

A calibration slope near 1.0 indicates good agreement between predicted and observed risk; the Brier score summarizes overall accuracy (lower is better). Values are optimism-corrected over 500 bootstrap resamples. Abbreviations: CALLY, CRP-albumin-lymphocyte index; GINI, Global Immune-Nutrition-Inflammation Index; NLR, neutrophil-to-lymphocyte ratio.

Table S6b. Internally validated calibration with 95% confidence intervals.

Model	Calibration slope (95% CI)	Calibration intercept (95% CI)	Brier score (95% CI)
Age + NLR	1.00 (0.66–1.54)	0.00 (-0.335–0.464)	0.177 (0.177–0.186)
Age + CALLY	1.00 (0.65–1.48)	0.00 (-0.319–0.392)	0.180 (0.18–0.189)
Age + GINI	1.00 (0.67–1.5)	0.00 (-0.339–0.499)	0.178 (0.178–0.188)

Bootstrap percentile confidence intervals over 500 resamples. A slope near 1.0 and an intercept near 0 indicate good agreement between predicted and observed risk; the wide slope intervals show that calibration is estimated imprecisely in a cohort of this size. Abbreviations as in Table S9.

Supplementary Methods (addendum). Retrospective reconstruction of the BISAP score.

BISAP was not recorded prospectively and was reconstructed for this study from the admission record. The five standard components were derived as follows: blood urea nitrogen above 25 mg/dL from the admission laboratory panel; age above 60 years from demographic data; pleural effusion from the structured radiology report; systemic inflammatory response syndrome from admission vital signs together with the white-cell count; and impaired mental status from the clinical narrative. Components without available source data were scored as absent, and the number of assessable criteria was recorded for each patient: five of five in 152 patients (63.9%), four of five in 83 (34.9%), and three of five in 3 (1.3%). Component positivity in the analytic cohort was: blood urea nitrogen above 25 mg/dL in 26 (10.9%), age above 60 years in 86 (36.1%), pleural effusion in 16 (6.7%), and systemic inflammatory response syndrome or impaired mental status in 33 (13.9%). Respiratory rate was documented in only a small minority of records and mental status was frequently unrecorded; temperature was missing in 73.9% and heart rate in 62.6% of the analytic cohort. The score is therefore expected to be mildly underestimated, which explains the low observed distribution (maximum observed 4; only nine patients scored at least 3). All incremental analyses accordingly refer to a retrospectively reconstructed BISAP score.

Table S7. Patient-clustered (GEE) association of the three leading indices with the composite outcome.

Index	Adjusted OR per 1-SD (95% CI)	p	Admissions (n)
NLR	2.25 (1.67–3.01)	<0.001	335
CALLY	0.42 (0.29–0.59)	<0.001	335
GINI	2.45 (1.75–3.42)	<0.001	335

Generalized estimating equations (GEE) with an exchangeable working correlation and clustering by patient, adjusted for age, using all qualifying admissions with a complete index panel (335 admissions). One otherwise-eligible admission with PIV = 0 (undefined logarithm) was excluded. These estimates assess association and do not validate the ROC ranking. Abbreviations: CI, confidence interval; GEE, generalized estimating equations; CALLY, CRP-albumin-lymphocyte index; GINI, Global Immune-Nutrition-Inflammation Index; NLR, neutrophil-to-lymphocyte ratio; OR, odds ratio; PIV, pan-immune-inflammation value; ROC, receiver operating characteristic; SD, standard deviation.

Table S8. Distribution and observed composite-outcome rate by the exploratory bedside integer score.

Risk score	n	Composite events	Observed rate (%)
0	62	8	12.9
1	72	13	18.1
2	70	32	45.7
3	18	10	55.6
4	16	10	62.5

Exploratory score. One point for age 65 to 74 years and two points for age of at least 75 years; one point for the middle CALLY tertile and two points for the lowest CALLY tertile (thresholds 0.0555 and 0.3018 in this cohort). Higher scores indicate higher risk. Apparent AUC 0.722 (95% CI 0.654 to 0.790); nested-bootstrap-corrected AUC (re-estimating the tertile thresholds in each resample) 0.722. Because the selection of CALLY among the leading indices was itself data-driven and is not repeated in the bootstrap, the true optimism may be slightly larger; the score is exploratory and not a validated instrument. Abbreviations: AUC, area under the receiver operating characteristic curve; CALLY, CRP-albumin-lymphocyte index; CI, confidence interval.

Additional Tables

Table S9. Discrimination of the simplest constituent analytes entered alone, compared with the composite indices.

Predictor (age-adjusted)	AUC (95% CI)	OR per 1-SD (95% CI)
CRP	0.702 (0.628–0.776)	1.98 (1.42–2.76)
Albumin	0.623 (0.544–0.702)	0.62 (0.46–0.84)
Lymphocyte	0.703 (0.629–0.778)	0.5 (0.36–0.67)
Neutrophil	0.683 (0.609–0.757)	2.02 (1.47–2.76)
Calcium	0.650 (0.573–0.728)	0.62 (0.47–0.83)
NLR (composite index)	0.737 (0.667–0.808)	see Table 4
CALLY (composite index)	0.735 (0.664–0.806)	see Table 4
GINI (composite index)	0.734 (0.661–0.806)	see Table 4

All models adjusted for age. Head-to-head DeLong comparisons of composite indices against single analytes: CALLY vs CRP p = 0.014; CALLY vs albumin p = 0.012; NLR vs CRP p = 0.331; NLR vs albumin p = 0.025; GINI vs CRP p = 0.130; GINI vs albumin p = 0.020. Abbreviations: AUC, area under the receiver operating characteristic curve; CALLY, CRP-albumin-lymphocyte index; CI, confidence interval; CRP, C-reactive protein; GINI, Global Immune-Nutrition-Inflammation Index; NLR, neutrophil-to-lymphocyte ratio; OR, odds ratio; SD, standard deviation.

Table S10. Sensitivity analysis using the harder endpoint of ICU admission or death (15 events).

Index	Age-adjusted AUC (95% CI)	OR per 1-SD	p
NLR	0.828 (0.725–0.930)	3.12	<0.001
CAR	0.778 (0.654–0.903)	2.47	0.018
PIV	0.738 (0.594–0.881)	1.97	0.013
PNI	0.803 (0.682–0.924)	0.44	<0.001
HALP	0.730 (0.556–0.905)	0.5	0.005
CALLY	0.816 (0.713–0.919)	0.33	0.002
CCR	0.771 (0.643–0.899)	2.4	0.022
GINI	0.779 (0.673–0.884)	2.52	0.004

Endpoint: in-hospital death or ICU admission during the index admission (15 events among 238 patients). Discrimination was numerically higher than for the primary composite outcome for every index, but the small number of events yields wide confidence intervals and these estimates are exploratory. Abbreviations as in Table S9.

Table S11. Performance of each index within its own largest available cohort.

Index	n	Age-adjusted AUC (95% CI)	OR per 1-SD	AUC in common cohort (n = 238)
NLR	325	0.719 (0.658–0.780)	2.19	0.737
CAR	245	0.718 (0.647–0.790)	2.09	0.710
PIV	325	0.697 (0.632–0.761)	1.85	0.708
PNI	245	0.709 (0.638–0.779)	0.46	0.704
HALP	245	0.685 (0.604–0.766)	0.5	0.686
CALLY	242	0.742 (0.672–0.812)	0.41	0.735
CCR	298	0.702 (0.637–0.768)	1.98	0.709
GINI	242	0.743 (0.672–0.814)	2.52	0.734

Each index was recomputed among all index admissions in which that index alone could be calculated, rather than restricting to the common complete-case cohort. Estimates were essentially unchanged, arguing against major distortion introduced by complete-case selection. Abbreviations as in Table S9.

Table S12. Discrimination stratified by documented versus undefined etiology.

Index	Documented etiology (n = 96, 37 events)	Undefined etiology (n = 142, 36 events)	Interaction p
NLR	0.660 (0.541–0.778)	0.795 (0.710–0.880)	0.119
CALLY	0.679 (0.558–0.800)	0.781 (0.698–0.864)	0.243
GINI	0.627 (0.503–0.751)	0.812 (0.733–0.891)	0.039

Age-adjusted AUCs within each stratum. The interaction term tests whether the index-outcome association differs by etiology stratum. Interactions were not corrected for multiple testing and are hypothesis-generating. Abbreviations as in Table S9.

Table S13. Incremental discrimination of all eight indices beyond the retrospectively reconstructed BISAP score.

Model	AUC (95% CI)	ΔAUC (apparent)	ΔAUC (optimism-corrected)	LRT p
BISAP alone	0.631 (0.559–0.704)	NA	NA	NA
BISAP + NLR	0.746 (0.676–0.817)	+0.115	+0.112	<0.001
BISAP + CAR	0.725 (0.652–0.798)	+0.094	+0.089	<0.001
BISAP + PIV	0.720 (0.645–0.795)	+0.089	+0.085	<0.001
BISAP + PNI	0.725 (0.655–0.794)	+0.093	+0.084	<0.001
BISAP + HALP	0.710 (0.633–0.787)	+0.079	+0.070	<0.001
BISAP + CALLY	0.752 (0.681–0.822)	+0.121	+0.120	<0.001
BISAP + CCR	0.724 (0.651–0.797)	+0.093	+0.089	<0.001
BISAP + GINI	0.738 (0.666–0.809)	+0.107	+0.103	<0.001

Because age is one of the five BISAP components, age was not entered separately. Optimism correction used 300 bootstrap resamples. BISAP was reconstructed retrospectively and is probably underestimated in this cohort (only nine patients scored at least 3), so these comparisons should not be read as superiority over a prospectively applied severity score. Abbreviations: BISAP, Bedside Index for Severity in Acute Pancreatitis; LRT, likelihood-ratio test; other abbreviations as in Table S9.

Supplementary Note S1. Sensitivity analysis using an inclusive ICU definition

When the composite outcome was redefined using a broader (inclusive) ICU definition (25 ICU admissions instead of 15), 77 patients (32.4%) met the composite endpoint. The discriminatory performance and ranking of the inflammatory indices remained essentially unchanged, with NLR, CALLY, and GINI continuing to show the highest discrimination and no statistically significant differences between them.

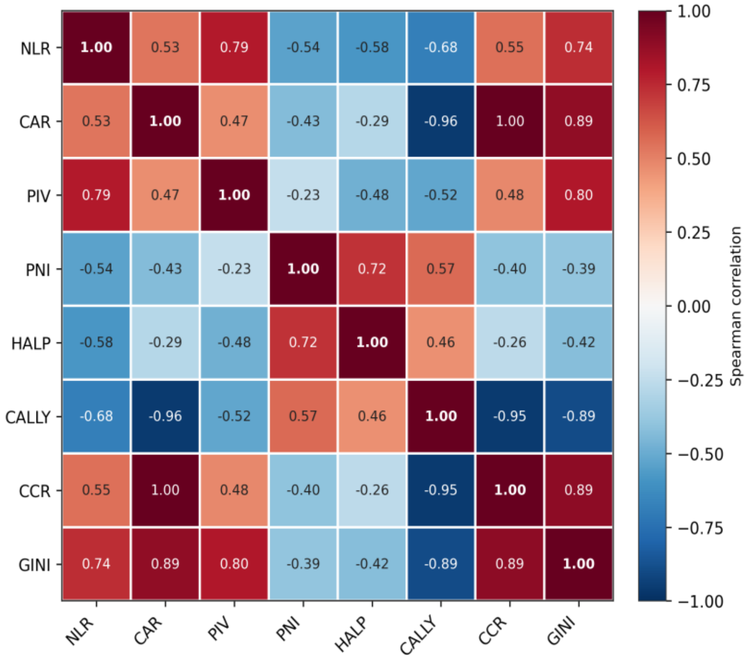

Figure S1. Spearman correlation matrix of the eight log-transformed indices.

Warm (red) cells denote positive correlation and cool (blue) cells negative correlation, with the numeric Spearman coefficient shown in each cell. CAR, CCR, and CALLY form a tightly correlated CRP/albumin cluster; GINI is strongly but not perfectly correlated with each of its components (CAR and PIV).

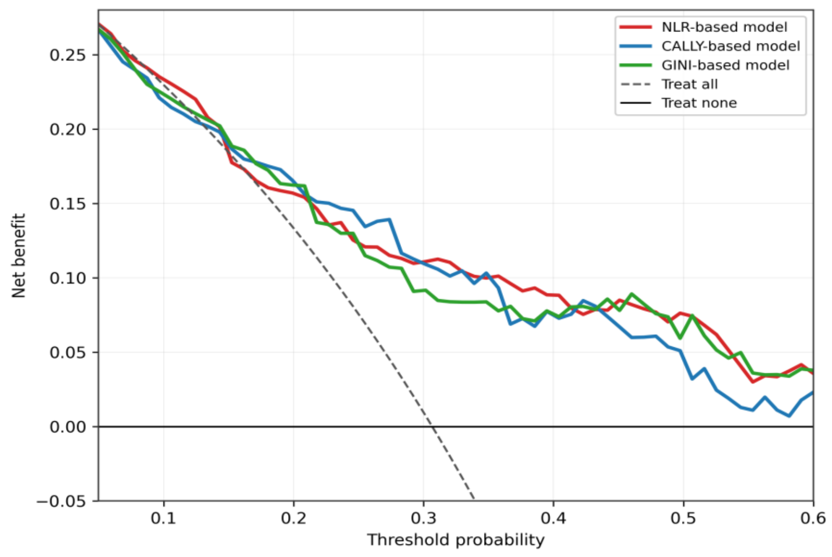

Figure S2. Decision-curve analysis of the three leading age-adjusted models (exploratory).

Net benefit across threshold probabilities for the age-plus-index models relative to the treat-all and treat-none strategies. Curves were derived and evaluated in the same cohort and are exploratory. Net benefit was concentrated at low-to-moderate thresholds relevant to a decision to intensify monitoring or seek earlier senior review.

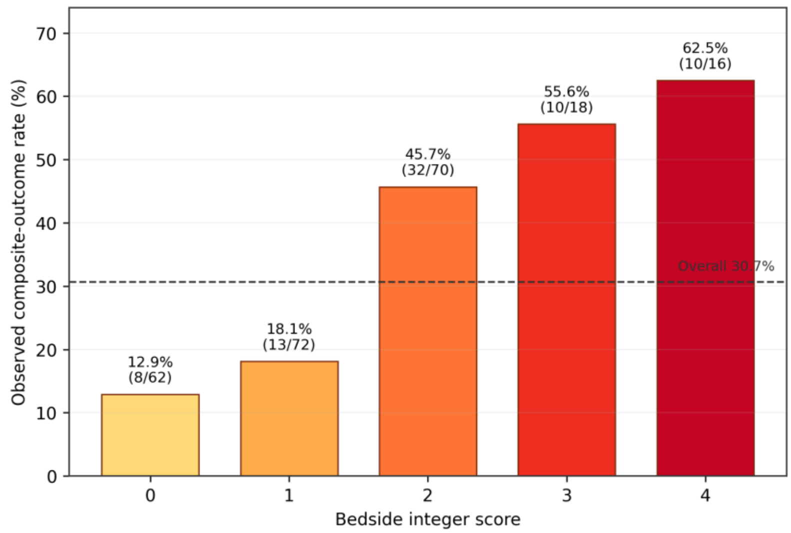

Figure S3. Observed composite-outcome rate by the exploratory bedside integer score (0 to 4).

Bar annotations give the observed rate and events over patients; the dashed line marks the overall composite-outcome rate (30.7%). This score is exploratory (see Table S8).
